# Uric acid in predicting the traumatic rhabdomyolysis induced acute kidney injury; a systematic review and meta-analysis

**DOI:** 10.1186/s12882-024-03509-x

**Published:** 2024-03-05

**Authors:** Saeed Safari, Mohammadreza Ghasemi, Mahmoud Yousefifard, Alireza Ghasemi, Iraj Najafi

**Affiliations:** 1 Men’s Health and Reproductive Health Research Center, Shahid Beheshti University of Medical Sciences, Tehran, Iran; 2 Emergency Department, Shohadaye Tajrish Hospital, Shahid Beheshti University of Medical Sciences, Tehran, Iran; 3 Physiology Research Center, Iran University of Medical Sciences, Tehran, Iran; 4 Student Research Committee, Alborz University of Medical Sciences, Karaj, Iran; 5 Nephrology Department, Shariati Hospital, Tehran University of Medical Sciences, Tehran, Iran

**Keywords:** Acute kidney injury, Rhabdomyolysis, Uric acid, Meta-analysis

## Abstract

**Objective:**

The objective of this systematic review and meta-analysis was to assess the value of uric acid in predicting acute kidney injury caused by traumatic rhabdomyolysis.

**Methods:**

The search was conducted in MEDLINE, Scopus, Embase and Web of Science until November 1, 2023. Based on the inclusion and exclusion criteria, the articles were included by two independent researchers. Data regarding study design, patient characteristics, number of patients with and without AKI, mean and SD of uric acid and prognostic characteristics of uric acid were extracted from relevant studies. STATA version 17.0 was used to compute pooled measures of standardized mean differences, odds ratios, and diagnostic accuracy. I2 and chi-square tests were used to assess heterogeneity between studies.

**Results:**

We found 689 non-redundant studies, 44 of them were potentially relevant. Six articles met the inclusion criteria and were included in the review. The results of the meta-analysis confirmed that there was a significant correlation between serum uric acid levels and the occurrence of AKI (SMD = 1.61, 95% CI = 0.69 to 2.54, I2 = 96.94%; *p* value = 0.001). There were no significant publication biases.

**Conclusion:**

According to this meta-analysis, uric acid levels could be considered as a predictor of acute kidney injury following traumatic rhabdomyolysis.

**Supplementary Information:**

The online version contains supplementary material available at 10.1186/s12882-024-03509-x.

## Introduction

Rhabdomyolysis (RM) is a clinical syndrome distinguished by the disruption of structural integrity in skeletal muscle cells, leading to the release of intracellular constituents into the extracellular space. Trauma, immobilization, sepsis, and surgery are the most frequently reported causes of RM [1–3]. In the case of rhabdomyolysis, acute kidney injury (AKI) is a tragic yet preventable complication. Patients with RM are reported to have a 13–50% chance of developing AKI, which is associated with approximately 30% mortality increase [4]. Patient outcomes are worse in RM patients with AKI, which increases medical burden. Therefore, prevention and early diagnosis of AKI improve prognosis for these patients [5–8].

A severe physical injury or trauma can cause traumatic rhabdomyolysis, which is characterized by rapid breakdown of skeletal muscle tissue [9]. When there is significant muscle compression or crush injuries, such as those sustained in car accidents, crush syndrome, sever burns, compartment syndrome, or natural disasters, this condition may occur [10]. The symptoms of traumatic rhabdomyolysis include muscle pain, weakness, dark urine (due to myoglobinuria), and, in severe cases, renal dysfunction [11]. By hydration and other appropriate measures, and prompt medical intervention, the underlying trauma can be managed, complications prevented, and renal function supported [12]. A mass disaster such as an earthquake will result in a large number of traumatic rhabdomyolysis patients. Management of these patients will be challenging given the special circumstances following the disaster and the shortage of equipment and resources [13].

Furthermore, rhabdomyolysis can present with a variety of symptoms, from asymptomatic to multiple organ failure [14]. In both the treatment and logistic aspects of triaging at-risk patients, it is of utmost importance to identify potential effective factors of rhabdomyolysis-induced complications such as AKI. Several studies have examined clinical and laboratory factors and developed a limited number of clinical prediction rules [13–15].

Uric acid(UA) is produced by purine metabolism in the body. During high cell turnover states like hemolysis, rhabdomyolysis, and tumor lysis, uric acid levels can increase [16]. Increased serum uric acid levels have been shown to be associated with kidney disease. Despite uric acid’s intrinsic antioxidant properties, studies have found that increased levels of uric acid promote oxidative stress, inflammation, fibrosis pathways, and endothelial dysfunction [17–20]. Excessive uric acid increases the risk of acute kidney injury [21], impairs the contractile function of intraglomerular mesangial cells [22], and causes damage to mesangial and proximal tubule epithelial cells through Toll like receptor 4-dependent activation of NLRP3 and IL-1β [23]. Hyperuricemia was also shown to be an independent risk factor for chronic kidney disease via injury of the endothelial cells and release of the high mobility group box 1 protein (HMGB1), stimulating Toll-like receptors (TLR) to induce pro-inflammatory and chemotactic cytokines, vascular smooth muscle proliferation, and activation of the NLRP3 inflammasome [24]. Furthermore, uric acid may accumulate in the kidneys, resulting in stone formation [25].

Studies have reported that UA can act as a predictive factor in a number of diseases. Some of them suggest that in rhabdomyolysis patients, elevated UA levels may be associated with an increased risk of AKI. However, whether UA is a prognostic factor in rhabdomyolysis-induced AKI remains controversial [26–30].

Therefore, this study evaluated the value of uric acid in predicting traumatic rhabdomyolysis-induced AKI by meta-analyzing existing studies.

## Method

### Search strategy

The preferred reporting items for systematic reviews and meta-analysis (PRISMA) guideline was followed in the present study [31]. The keywords were selected using three strategies: MeSH terms, Emtree terms, and consultation with experts. We conducted a systematic search through PubMed/MEDLINE, Scopus, Web of Science (WOS) and Embase databases for relevant articles published since inception until November 1, 2023 (search strategy of all databases is reported in Supplementary Table 1).

Scopus was used to identify additional documents in the gray literature, such as conference abstracts. A hand search of the reference lists of relevant studies was conducted to find additional articles and unpublished data.

Our systematic review question was formulated as follows [32]: The population (P) were patients with traumatic-rhabdomyolysis, the index test (I) was serum uric acid level(mg/dl), the reference test (R) was any valid definition of AKI using serum creatinine level, glomerular filtration rate (GFR), Kidney Disease Improving Global Outcomes (KDIGO) guidelines or risk, injury, failure, loss of kidney function, and end-stage kidney disease (RIFLE) criteria.

A protocol for this study is registered in the International Prospective Register of Systematic Reviews (PROSPERO) with the identifier CRD42023460189.

### Eligibility criteria and screening method

The inclusion criteria were as follows: traumatic-rhabdomyolysis patients as a population; measurement of serum uric acid level; prediction of AKI incidence using GFR, serum creatinine level, KDIGO or RIFLE criteria; and having a control group (without AKI). Analyses were conducted both retrospectively and prospectively. We excluded studies without a control group (non-AKI patients), in non-traumatic patients, without assessments of serum uric acid, animal studies, case reports, case series, reviews, systematic reviews, letters to editors, editorials and protocols. There was no restriction on the participants’ age. No language restriction is considered. The screening and selection process was carried out independently by two authors (M.G. and A.G). Disagreements were resolved either through discussion or by expert reviewers (M.Y. and S.S).

### Data extraction and risk of bias assessment

Both authors (M.G and A.G.) independently extracted data, including the name of the first author, the publication year, the study design, the country, the age of the population, the definition of AKI, the total sample size, the number of participants with AKI and those without, and the mean and standard deviation (SD) of uric acid levels in AKI and non-AKI patients. The data extraction sheet is summarized in Table 1. Whenever data in an article could not be extracted, its corresponding author was contacted. Results presented as graphs were extracted using web plot digitizer [33].

**Table 1:** Characteristics of the included studies

First author	Year	Design	Location	Patients(n)		Age ^a^	Male (%)	AKI definition	Etiology of trauma
				AKI+	AKI-				
Yoo	2021	Cross-sectional	South Korea	99	781	11.1(6.8)	72.0	KIDGO criteria	All cause trauma
Galeiras	2016	Cross-sectional	Spain	57	46	53.1(18.5)	80.5	Creatinine ≥ 1.2	Traumatic SCI patients
Omrani	2020	Cross-sectional	Iran	10	216	41.6(24.1)	39.8	Creatinine ≥ 1.6	Kermanshah earthquake
Sever	2011	Cross-sectional	Turkey	261	9	NR	NR	RIFLE criteria	Marmara earthquake
Spampinato	2007	Cross-sectional	Italy	5	5	NR	NR	Nr	All cause trauma
Najafi	2008	Cross-sectional	Iran	94	1347	NR	52.0	Creatinine ≥ 1.6	Bam earthquake

AKI acute kidney injury, NR not reported, KIDGO kidney disease improving global outcom, RIFLE risk, injury, failure, loss of kidney function, and end-stage kidney disease, SCI spinal cord injury. ^a^ Data was presented as mean (standard deviation)

The risk of bias was assessed by the two authors (M.G. and A.G) independently, using the QUADAS-2 checklist [34, 35]. The differences between the two were resolved through discussion or by expert reviewers (M.Y. and S.S).

The QUADAS-2 includes two sections: The risk of bias (ROB) and applicability. The risk of bias section includes four domains, such as patient selection, index test, reference standard and flow and timing. There are three domains in the applicability section, including patient selection, index test, and reference standard. All domains are judged as “low” for answer yes, “high” for answer no, or unclear. Then, studies were classified according to the following categories: low risk of bias and low concern regarding applicability (when all domains were low risk) and at risk of bias or as having concerns regarding applicability (when at least one domain was high risk or unclear).

### Statistical analysis

The statistical analyses were conducted using Stata 17.0 (StataCorp LLC, College Station, TX, USA). We calculated a standardized mean difference (SMD) with a 95% confidence interval (95% CI) for each sample, and then pooled them to calculate an overall effect size. At least three separate analyses had to be reported for meta-analysis to be performed.

To compensate for methodological heterogeneities between studies, such as differences in population and methodology, a random-effects model was chosen. The I2 and chi-square tests were used to assess statistical heterogeneity between studies.

## Results

### Study characteristics

**Fig. 1:**
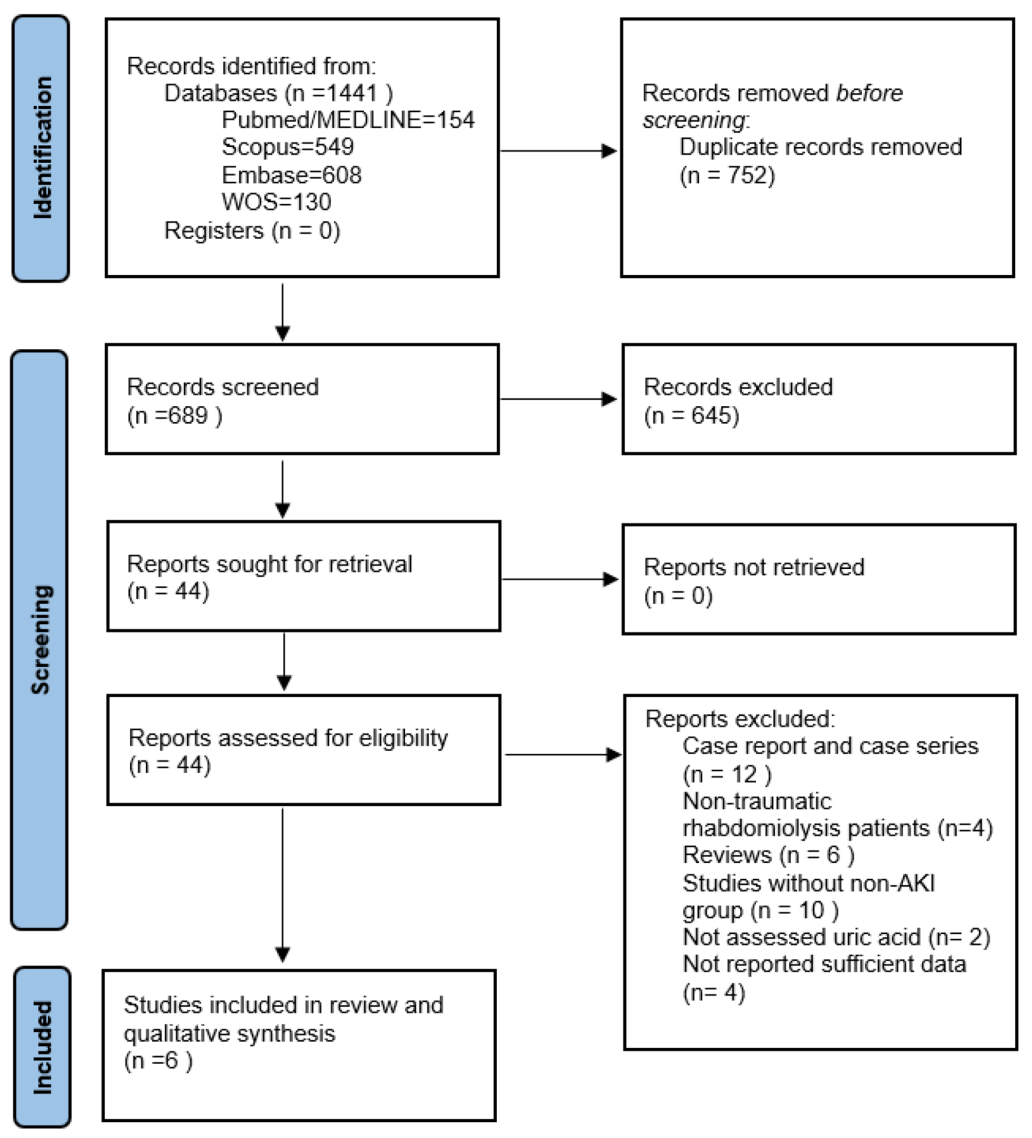
PRISMA flow diagram of the literature search and study selection process

The study flowchart is shown in Fig. 1. After removing duplicates from 1441 publications, 689 titles and abstracts were screened, and 44 potentially relevant articles were selected for full-text evaluation based on the inclusion and exclusion criteria.

Ultimately, six observational studies published between 2007 and 2021 were eligible for inclusion. Through forwarding and backward citation tracking of eligible studies, no additional articles were found. Table 1 summarizes the main characteristics of the included studies.

The studies were all cross-sectional. Four studies were conducted in Asia and two in Europe. In all studies, both sexes were included. The etiology of rhabdomyolysis was trauma in all studies. Three of the studies involved earthquake victims. AKI was defined in one study using RIFLE criteria, one using KIDGO criteria, two studies using creatinine values greater than 1.6 mg/dL in the first three days after admission, and in one study AKI definition was not mentioned.

In all six studies, mean serum uric acid levels were compared between participants with and without AKI [13, 15, 29, 30, 36, 37]. One study presents its findings as an OR, two studies present their findings as ROC curves [13, 15, 30]. There was no presentation of sensitivity or specificity in any of the studies. The meta-analyses were therefore limited to standardized mean differences.

### Comparing serum uric acid levels of patients with and without AKI

In these studies, 526 AKI + cases and 2404 AKI- cases were included. The average age of these patients was 46.03 ± 19.37 years, and 68.8% of them were male.

**Fig. 2:**
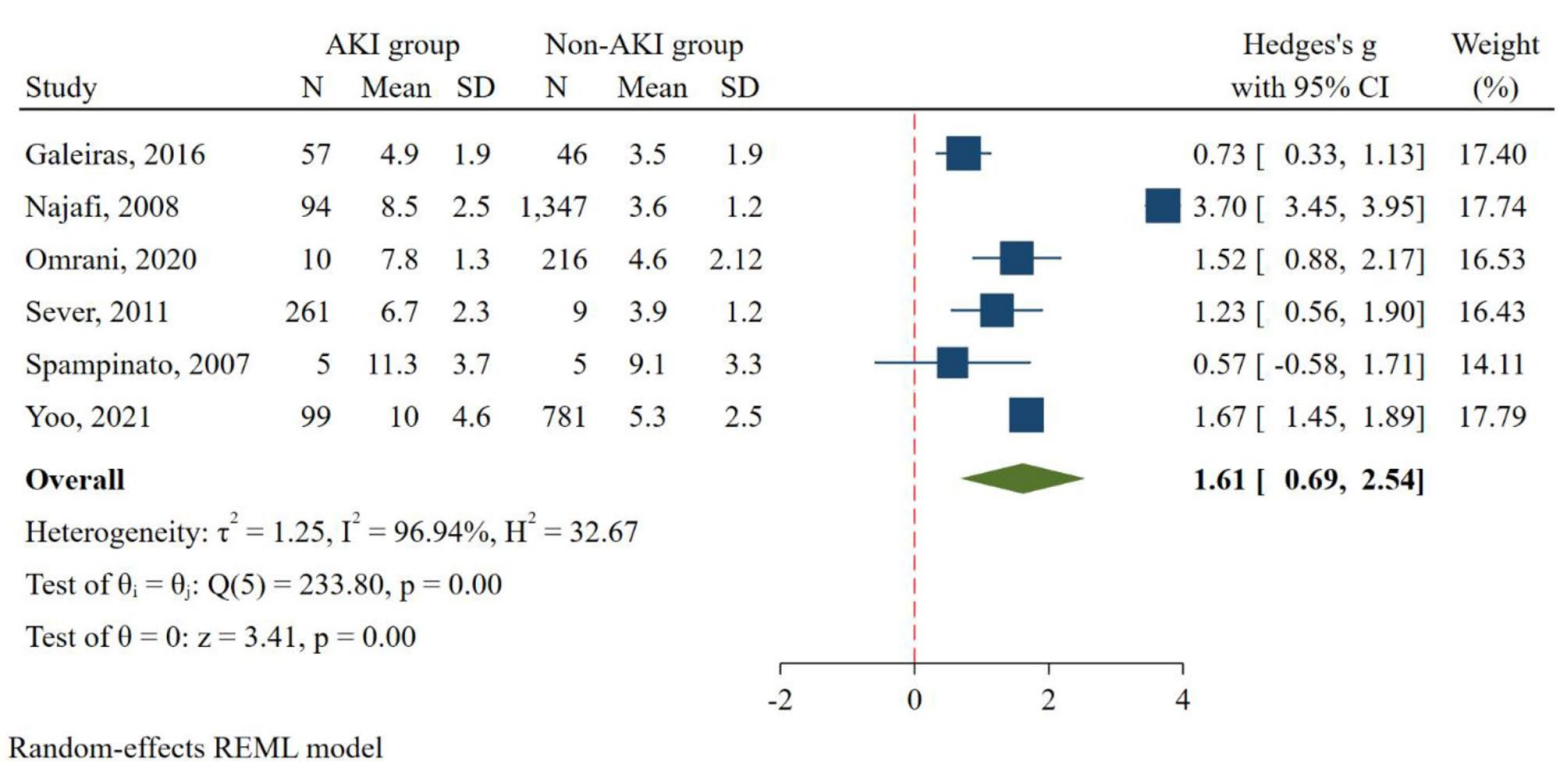
Forest plot (random-effects model) depicting the association of serum uric acid and occurrence of acute kidney injury

Comparing pooled standardized mean differences (SMD) showed a significant correlation between serum uric acid level and AKI occurrence in traumatic rhabdomyolysis patients (SMD = 1.61, 95% CI: 0.69 to 2.54, I_2_ = 96.94%; *p* value = 0.001). the mean and standard deviation of uric acid level in AKI and non-AKI group was 7.03 ± 2.12 and 4.25 ± 1.11 respectively. The result of the meta-analysis is presented in Fig. 2.

### Risk of bias assessment

Based on QUADAS-2, five studies were categorized as “high” and one as “low” risk of bias (Table 2). Two studies included did not use random selection for participants [36, 38]. Reference standard appropriate for correctly classifying target conditions in all studies. As well, index test results were interpreted without knowing the reference standard results.

**Table 2:** Detailed results of the risk of bias assessment of the included studies based on the QUADAS-2

Study	Risk OF Bias					Applicability Concern			
	Patient Selection	Index Test	Reference Standard	Flow & Timing	Overall Score	Patient Selection	Index Test	Reference Standard	Overall Score
Galeiras, 2016	Low	Low	Low	High	High	High	Low	Low	High
Najafi, 2008	Low	Low	Low	High	High	Low	Low	Low	High
Omrani, 2020	Low	Low	Low	High	High	Low	Low	Low	High
Sever, 2011	High	Low	Low	High	High	High	Low	Low	High
Spampinato,2007	High	Low	Low	Low	High	High	Low	Low	High
Yoo, 2021	Low	Low	Low	Low	Low	Low	Low	Low	Low

### Publication bias

**Fig. 3:**
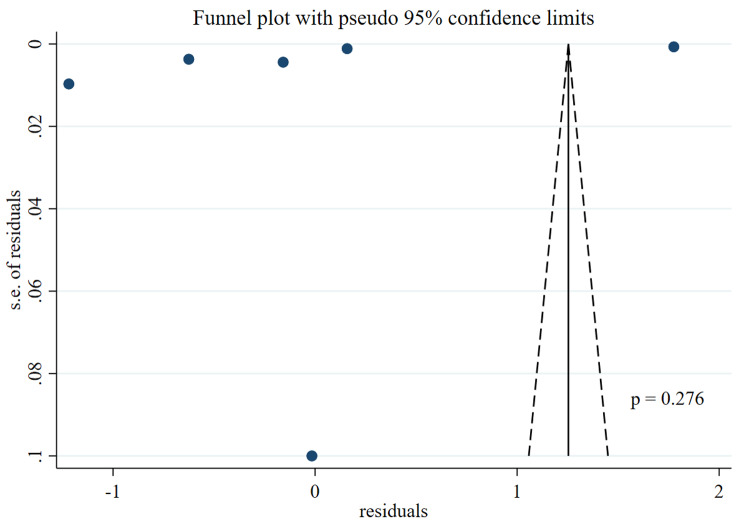
Publication bias assessment. s.e: standard error

Both funnel plots and Eggers tests were used to assess publication bias (Fig. 3). There was an assymetria in funnel plot, however, the egger test did not detect any significant publication bias (*p* = 0.2766).

## Discussion

This meta-analysis was the first to examine the association between serum uric acid levels and acute kidney injury following traumatic rhabdomyolysis. Our results showed a significant correlation between serum uric acid levels and AKI occurrence. This suggests that uric acid levels may be a potential biomarker for predicting AKI following traumatic rhabdomyolysis. In order to identify the proper cutoff point, further research is needed. Furthermore, it is crucial for researchers to report sensitivity, specificity, OR, and AUC in order to facilitate meta-analyses of these items.

Acute kidney injury (AKI) is an important complication of RM, which is associated with high morbidity and mortality. Through early and aggressive fluid therapy, AKI can be prevented in this condition. Even so, logistics supplies are often insufficient for fluid therapy in disaster situations. To manage AKI successfully, patients at risk must be distinguished from those not at risk [39].

Items that can help predict or identify patients at risk for AKI, or rules of thumb that can identify those who are at risk, would be very helpful. To make these items easy to use in emergency situations, simple clinical or biochemical parameters should be used. Several laboratory values have already been tested to identify a possible association with AKI [40–42].

When interpreting our results, it is important to keep the following in mind:

An important limitation of this study is the meta-analysis of observational studies. Observational designs are limited in nature and cannot assess all confounding factors, and therefore cannot prove causal relationships effectively.

Second, a crude SMD was used in this study due to insufficient data regarding adjusted effect sizes. Hence, various uncontrolled factors such as age, sex, comorbidities, duration of trauma to admission and unknown factors may confound the observed positive relationship between uric acid and AKI.

Third, we were unable to conduct meta-analyses on OR, sensitivity, specificity, and AUC due to insufficient data.

Although efforts were made to make the studies similar from a methodology perspective, there was heterogeneity between the studies. There was some variation between articles regarding the definition of AKI and the cut-point for creatin kinase for RM. In addition, Yoo et al. study was conducted on children (aged one month to 18 years) [30].

In addition, given the low number of eligible studies, subgroup analysis was not possible in terms of methodology.

Regarding the asymmetry in our funnel plots, Egger et al. have mentioned that asymmetry in funnel plots can be caused by several factors: publication bias, poor methodological quality, the presence of substantial heterogeneity, selective outcome reporting, sampling variations, and by chance. Consequently, publication bias might not be the only reason for the observed asymmetry [43].

In this meta-analysis, all six studies were in some way heterogeneous, so it was difficult to draw solid conclusions. This could be due to the limited relevant studies on this topic. Additionally, most of the studies didn’t report OR, sensitivity, specificity, and AUC, so meta-analysis was not possible on these items. Thus, to help reach a conclusive conclusion regarding uric acid’s ability to predict AKI caused by traumatic-rhabdomyolysis, more comprehensive and original research is needed.

## Conclusion

This meta-analysis shows that uric acid levels could be considered as a predictor of AKI following traumatic rhabdomyolysis. The results of these studies provide evidence for other researchers and clinicians to pay closer attention to this item in patients with traumatic rhabdomyolysis in order to prevent AKI.

### Electronic supplementary material

Below is the link to the electronic supplementary material.

Supplementary Material 1

## Data Availability

No datasets were generated or analysed during the current study.
