# Supplementary material for: Uric acid in predicting the traumatic rhabdomyolysis induced acute kidney injury; a systematic review and meta-analysis
Source: BMC Nephrol. 2024 Mar 5;25:82. doi: 10.1186/s12882-024-03509-x (PMC10916315; doi:10.1186/s12882-024-03509-x)
Supplement: Supplementary file 1 — Supplementary Material 1 [file 12882_2024_3509_MOESM1_ESM.docx]

**Medline (via pubmed)**

1. "Rhabdomyolysis"**[mh] OR** "Myoglobinuria"**[mh] OR** "Compartment Syndromes"**[mh] OR** "Anterior Compartment Syndrome"**[mh] OR** "Chronic Exertional Compartment Syndrome"**[mh] OR** "Popliteal Artery Entrapment Syndrome"**[mh] OR** "Crush Injuries"**[mh] OR** "Crush Syndrome"**[mh] OR** "Soft Tissue Injuries"**[mh] OR** Rhabdomyolysis**[tiab] OR** rhabdomyolyses**[tiab] OR** Bywater’s syndrome**[tiab] OR** Bywaters syndrome**[tiab] OR** myoglobinurias**[tiab] OR** myoglobinuria**[tiab] OR** myoglobulinuria**[tiab] OR** myohemoglobinuria**[tiab] OR** myohaemoglobinuria**[tiab] OR** compartment syndrome**[tiab] OR** compartment syndromes**[tiab] OR** Anterior Tibial Syndrome**[tiab] OR** Anterior Tibial Syndromes**[tiab] OR** Popliteal Artery Entrapment**[tiab] OR** Popliteal Artery Entrapments**[tiab] OR** Crush Injury**[tiab] OR** crush injuries**[tiab] OR** crush trauma**[tiab] OR** crushing injury**[tiab] OR** crushing trauma**[tiab] OR** Crush Syndrome**[tiab] OR** Crush Syndromes**[tiab] OR** skeletal muscle damage**[tiab] OR** muscle trauma**[tiab] OR** Soft Tissue Injury**[tiab] OR** Soft Tissue Injuries**[tiab] OR** muscle contusion**[tiab]**
2. Uric Acid**[mh] OR** hyperuricemia**[mh] OR** Uric Acid**[tiab] OR** Urate**[tiab] OR** Hyperuricacidaemia**[tiab] OR** Hyperuricacidemia**[tiab] OR** Hyperuricaemia**[tiab] OR** hypouricacidaemia**[tiab] OR** hypouricaemia**[tiab] OR** hyperuricemia**[tiab] OR** Hypouricemia**[tiab]**
3. **#1 AND #2**

**Embase:**

1. 'Rhabdomyolysis'**/exp OR** 'crush trauma'**/exp OR** 'crush syndrome'**/exp OR** 'Soft Tissue Injury'**/exp OR** 'compartment syndrome'**/exp OR** 'muscle contusion'**/exp OR** 'myoglobinuria'**/exp OR** 'rhabdomyolysis'**:ab,ti OR** **'**rhabdomyolyses**':ab,ti OR '**Bywaters syndrome**':ab,ti OR '**myoglobinurias**':ab,ti OR '**myoglobinuria**':ab,ti OR '**myoglobulinuria**':ab,ti OR '**myohemoglobinuria**':ab,ti OR '**myohaemoglobinuria**':ab,ti OR '**compartment syndrome**':ab,ti OR '**compartment syndromes**':ab,ti OR '**Anterior Tibial Syndrome**':ab,ti OR '**Anterior Tibial Syndromes**':ab,ti OR '**Popliteal Artery Entrapment**':ab,ti OR '**Popliteal Artery Entrapments**':ab,ti OR '**Crush Injury**':ab,ti OR '**crush injuries**':ab,ti OR '**crush trauma**':ab,ti OR '**crushing injury**':ab,ti OR '**crushing trauma**':ab,ti OR '**Crush Syndrome**':ab,ti OR '**Crush Syndromes**':ab,ti OR '**skeletal muscle damage**':ab,ti OR '**muscle trauma**':ab,ti OR '**Soft Tissue Injury**':ab,ti OR '**Soft Tissue Injuries**':ab,ti OR '**muscle contusion**':ab,ti**
2. 'uric acid'**/exp OR** 'Urate'**/exp OR** 'uric acid blood level'**/exp OR** 'Hyperuricemia'**/exp OR** 'Hypouricemia'**/exp OR** 'Uric Acid'**:ti,ab OR** 'Urate'**:ti,ab OR** 'Hyperuricacidaemia'**:ti,ab OR** ' Hyperuricacidemia'**:ti,ab OR** ' Hyperuricaemia'**:ti,ab OR** 'hypouricacidaemia'**:ti,ab OR** 'hypouricacidemia'**:ti,ab OR** ' hypouricaemia'**:ti,ab OR** ' hypouricidemia'**:ti,ab OR** 'Hypouricemia'**:ti,ab OR** 'hyperuricemia'**:ti,ab**
3. **#1 AND #2**

**Scopus :**

1. **TITLE-ABS-KEY**(**"**rhabdomyolysis**" OR "**rhabdomyolyses**" OR "**Bywater’s syndrome**" OR "**Bywaters syndrome**" OR "**myoglobinurias**" OR "**myoglobinuria**" OR "**myoglobulinuria**" OR "**myohemoglobinuria**" OR "**myohaemoglobinuria**" OR "**compartment syndrome**" OR "**compartment syndromes**" OR "**Anterior Tibial Syndrome**" OR "**Anterior Tibial Syndromes**" OR "**Popliteal Artery Entrapment**" OR "**Popliteal Artery Entrapments**" OR "**Crush Injury**" OR "**crush injuries**" OR "**crush trauma**" OR "**crushing injury**" OR "**crushing trauma**" OR "**Crush Syndrome**" OR "**Crush Syndromes**" OR "**skeletal muscle damage**" OR "**muscle trauma**" OR "**Soft Tissue Injury**" OR "**Soft Tissue Injuries**" OR "**muscle contusion**"**)
2. **TITLE-ABS-KEY**("Uric Acid" **OR** "Urate" **OR** "Hyperuricacidaemia" **OR** " Hyperuricacidemia" **OR** " Hyperuricaemia" **OR** "hypouricacidaemia" **OR** "hypouricacidemia" **OR** " hypouricaemia" **OR** " hypouricidemia" **OR** "Hypouricemia" **OR** "hyperuricemia")
3. **#1 AND #2**

**Web of science :**

1. **TS=**(**"**rhabdomyolysis**" OR "**rhabdomyolyses**" OR "**Bywater’s syndrome**" OR "**Bywaters syndrome**" OR "**myoglobinurias**" OR "**myoglobinuria**" OR "**myoglobulinuria**" OR "**myohemoglobinuria**" OR "**myohaemoglobinuria**" OR "**compartment syndrome**" OR "**compartment syndromes**" OR "**Anterior Tibial Syndrome**" OR "**Anterior Tibial Syndromes**" OR "**Popliteal Artery Entrapment**" OR "**Popliteal Artery Entrapments**" OR "**Crush Injury**" OR "**crush injuries**" OR "**crush trauma**" OR "**crushing injury**" OR "**crushing trauma**" OR "**Crush Syndrome**" OR "**Crush Syndromes**" OR "**skeletal muscle damage**" OR "**muscle trauma**" OR "**Soft Tissue Injury**" OR "**Soft Tissue Injuries**" OR "**muscle contusion**"**)
2. **TS=**("Uric Acid" **OR** "Urate" **OR** "Hyperuricacidaemia" **OR** " Hyperuricacidemia" **OR** " Hyperuricaemia" **OR** "hypouricacidaemia" **OR** "hypouricacidemia" **OR** " hypouricaemia" **OR** " hypouricidemia" **OR** "Hypouricemia" **OR** "hyperuricemia")
3. **#1 AND #2**
